# The Durability of Zirconia/Resin Composite Shear Bond Strength using Different Functional Monomer of Universal Adhesives

**DOI:** 10.1055/s-0041-1736331

**Published:** 2021-12-13

**Authors:** Awiruth Klaisiri, Nantawan Krajangta, Niyom Thamrongananskul

**Affiliations:** 1 Division of Restorative Dentistry, Faculty of Dentistry, Thammasat University, Pathum Thani, Thailand; 2 Department of Prosthodontics, Faculty of Dentistry, Chulalongkorn University, Bangkok, Thailand

**Keywords:** resin composite, shear bond strength, universal adhesive, zirconia

## Abstract

**Objective** This study examined the effectiveness of different functional monomers in universal adhesives on zirconia/resin composite bond strength both before and after thermocycling. Four universal adhesives (G-premio bond universal, GPU; Clearfil Tri-S bond universal, CTB; Optibond Universal, OBU; Tetric N-bond universal; TNU), one adhesive (single bond 2; SB2), and one ceramic primer (Clearfil ceramic primer plus, CCP) were used in this study.

**Materials and Methods** Zirconia discs were prepared and embedded in acrylic. Specimens were polished and sandblasted with alumina. The specimens were randomly divided into two groups (24 hours and the thermocycled), and each group was divided into six subgroups ( *n* = 10), according to zirconia surfaces treatments: no Tx, CCP + SB2, GPU, CTB, OBU, TNU. An Ultradent mold was located on top of the treated zirconia surface. The resin composite was filled into the mold and then light-cured. A universal testing device was used to determine the shear bond strength.

**Statistical Analysis** The data were statistically analyzed using one-way ANOVA and Tukey's test.

**Results** After water storage for 24 hours, the shear bond strengths were GPU > CCP + SB2 = CTB = OBU = TNU > no Tx ( *p* < 0.05). After thermocycling, the shear bond strengths were CCP + SB2 = GPU = CTB = TNU > OBU > no Tx ( *p* < 0.05).

**Conclusion** The universal adhesives containing 10-MDP exhibited the best performance in the shear bond strength of the zirconia/resin composite interface both before and after thermocycling.

## Introduction

In restorative dentistry and prosthodontics, zirconia has been widely used, particularly for inlays, onlays, crowns, bridges, and implant materials. Because of its biocompatibility, strength, and esthetics in dentistry, zirconia has increased in popularity. 1 Despite their high-success rate, the most common cause of zirconia restoration failure is chipping or fracture of the zirconia. 2 Resin composite repair methods reduce chair time and costs for patients. 3 4 5 Furthermore, intraoral repair has been suggested as a viable treatment alternative option if the indication and treatment procedures are proper. In cases of chipping or fracture of zirconia, the adhesion between resin composite and zirconia would influence the prognosis of the intraoral repair. The zirconia surface modification, including surface cleaning for micromechanical retention and surface modification for chemical adhesion on the zirconia, should be performed to improve the bond strength between resin composite and zirconia. 3

Universal adhesives have become a new trend in adhesive and restorative dentistry. It is further stated that universal adhesives can be used not only to bond to enamel and dentin but as adhesive primers on materials such as zirconia, silica-based ceramics, noble metals, base metals, and resin composites. The universal adhesives use phosphate and/or carboxylate monomers as their primary adhesive functional monomer. Acidic functional monomers are commonly utilized, including carboxylate monomers such as 4-methacryloyloxyethyl trimellitate anhydride (4-META) and phosphate monomers such as 10-methacryloyloxydecyl dihydrogen phosphate (10-MDP) or glycerol phosphate dimethacrylate (GPDM). These monomers have many positive attributes, including the potential to bond chemically to zirconia, 6 metals, 7 and tooth structures via the formation of nonsoluble calcium salts. 8 9

Thermocycling is a technique for simulating the restorations' artificial aging process. This technique gives information on the bonding resin and zirconia adhesive failure due to the dissimilar coefficients of thermal expansion between the adhesive and zirconia. The aging process has been demonstrated using a variety of thermocycling regimens, however, Gale et al have described that a cyclic procedure of 10,000 cycles per year may be enough to indicate restorative adhesive failure. 10

This laboratory study aimed to compare shear bond strength (SBS) of four universal adhesives G-premio bond universal (GPU), Clearfil Tri-S bond universal (CTB), Optibond Universal (OBU), Tetric N-bond universal (TNU) on the resin composite and zirconia interface both before and after thermocycling with standard control Clearfil ceramic primer plus (CCP) + single bond 2 (SB2) and negative control (no chemically surface modification).

## Materials and Methods

This was a randomized control group study using 120 fully sintered zirconia disc specimens (VITA YZ HT, VITA Zahnfabrik, Germany) 6.0 mm in diameter and 4.0 mm in thickness. At first, the specimens were embedded in polyvinyl chloride pipe with acrylic. The specimen's surfaces were polished with 600 grit silicon carbide abrasive paper (3M Wetordry Abrasive Sheet, 3M, MN, USA). The specimens were sandblasted with 50 µm aluminum oxide (Rocatec, 3M ESPE, St. Paul, MN, USA) perpendicularly in the zirconia surface (2.5 bars, 10 mm in distance, for 10 seconds) to create micromechanical retention. All specimens were then ultrasonically cleaned (Ultrasonic cleaner VI, Yoshida dental trade distribution Co., Tokyo, Japan) for 10 minutes in distilled water and then dried with oil-free air for 10 seconds from a triple syringe. The specimens were randomly divided into two groups (water storage for 24 hours and 10,000 cycles of thermocycling), and each group was divided into six subgroups ( *n* = 10) according to zirconia surface treatments: no Tx, CCP + SB2, GPU, CTB, OBU, and TNU. The predictor variable was adhesives/primer, which was a nominal scale (GPU, CTB, OBU, TNU, CCP, and SB2). The specimens treated by CCP + SB2, a standard adhesive for zirconia/resin composite repair, as standard controls, while negative controls were no chemically zirconia surface modification. Table 1 showed the types, brand names, manufacturers' details, lot numbers, and chemical composition of the adhesives/primers used in this study.

**Table 1:** Materials used in this study

Material	Composition
Clearfil ceramic primer plus (Kuraray Noritake Dental Inc., Okayama, Japan) Lot: 410043	10-MDP, ethanol, 3-trimethoxysilylpropyl methacrylate
Clearfil Tri-S bond universal (Kuraray Noritake Dental Inc., Okayama, Japan) Lot: 4K0025	10-MDP, Bis-GMA, HEMA, colloidal silica, ethanol, silane, sodium fluoride, camphoquinone, ethanol, water
Optibond universal (Kerr Corporation, California, USA) Lot: 6920782	GPDM, GDM, HEMA, dimethacrylate, acetone, ethanol
G-premio bond universal (GC Corporation, Tokyo, Japan) Lot: 1611221	10-MDP, 4-MET, HEMA, dimethacrylate, ethanol, acetone
Tetric N-bond universal (Ivoclar vivadent, AG, FL-9494 Schaan, Liechtenstein) Lot: X43844	10-MDP, 2-hydroxyethyl methacrylate, Bis-GMA, ethanol, 1, 10-decandiol dimethacrylate, camphorquinone, 2-dimethylaminoethyl methacrylate
Single bond 2 (3M ESPE, St. Paul, Minnesota, USA) Lot: N378816	Bis-GMA, HEMA, DMA, methacrylate functional copolymer, filler, photoinitiators, ethanol, water

Abbreviations: 10-MDP, methacryloyloxydecyl dihydrogen phosphate; 4-MET, 4-methacryloxyethyl trimellitic acid; Bis-GMA, bisphenol A-glycidyl methacrylate; DMA, dimethacrylate; GDM, 1,3-glycerol dimethacrylate; GPDM, glycerol phosphate dimethacrylate; HEMA, 2-hydroxyethyl methacrylate.

The samples were randomized into 12 subgroups ( *n* = 10). All of them, except standard and negative control, were treated with one adhesive according to their group using a microbrush, dried 10 seconds with oil-free air from a triple syringe, and then light-cured for 20 seconds (Elipar FreeLight2 LED Curing Light, 3M ESPE, MN, USA). The standard control samples were first conditioned with CCP, dried 10 seconds with oil-free air from a triple syringe, subsequently treated with SB2, dried again for 10 seconds in the same method, and finally light-cured for 20 seconds. The negative control samples were no chemically zirconia surface modification.

The Ultradent mold (Ultradent product, Inc., South Jordan, USA) 2.0 mm in diameter, and 2.0 mm in thickness was located on the zirconia pretreated surfaces to help to display the bonding region, filled with resin composite (Harmonize A4D shade, Kerr Corporation, California, USA), and then light-cured 40 seconds.

The bonded samples were stored for 30 minutes at room temperature and then were kept in distilled water at 37 °C in an incubator (Incubator, Humanlab instrument Co., Suwon, Korea) for 24 hours; one half was then tested (24 hours) and the other half was thermocycled (Proto-tech, Micoforce, Portland, OR, USA) for 10,000 cycles with different temperatures of 5 °C and 55 °C, with a reside time of 30 seconds in each bath and 5 seconds of a transfer time.

A universal testing equipment (AGS-X 500N, Shimadzu Corporation, Kyoto, Japan) was used to measure the SBS with an external load tested in the direction parallel to the zirconia/resin composite interface at 0.5 mm/min of crosshead speed. The SBS was determined by dividing the force at which bond failure appeared at the zirconia/resin composite interface.

Under a stereomicroscope (ML9300, Meiji Techno Co. Ltd., Saitama, Japan) with a magnification of x40, the fracture pattern was identified to quantify the postloading failure mode percentage at the fractured zirconia/resin composite interface. The fracture mechanism was classified into three different types: adhesive failure at the zirconia/resin composite interface, cohesive failure within the zirconia or resin composite substance, and mixed failure was a result of a mix of the two.

During the study period, a collecting form was used to collect data and recorded into a statistical database (SPSS, SPSS Inc., Chicago, IL, USA) for analysis. The statistical analysis results, using one-way ANOVA and Tukey's test, were computed as appropriate. *p* < 0.05 was used to determine statistical significance in all of the analyses.

## Results

The SBS values of all test groups are presented in Table 2 . After water storage for 24 hours, the SBS values of the specimens ranging from high to low were as follows: GPU > CCP + SB2 = CTB = OBU = TNU > no Tx ( *p* < 0.05). After thermocycling for 10,000 cycles, the SBS values were: CCP + SB2 = GPU = CTB = TNU > OBU > no Tx ( *p* < 0.05). Compared with water storage for 24 hours, the SBS values of the GPU, OBU and no Tx groups were lower after thermocycling for 10,000 cycles, but the SBS values of CCP + SB2, CTB, and TNU groups did not change significantly ( *p* > 0.05).

**Table 2:** The mean SBS values of samples (X [SD], *n* = 10)

Groups	SBS (MPa)	
	24 hours	10,000 TC
1. No Tx	7.27 (1.72) ^Aa^	3.29 (1.17) ^Ab^
2. CCP + SB2	19.15 (1.87) ^Ba^	18.48 (1.69) ^Ba^
3. GPU	25.77 (2.12) ^Ca^	17.93 (1.35) ^Bb^
4. CTB	19.37 (1.74) ^Ba^	18.88 (1.41) ^Ba^
5. OBU	19.62 (1.22) ^Ba^	12.75 (0.89) ^Cb^
6. TNU	18.62 (1.21) ^Ba^	18.38 (1.28) ^Ba^

Abbreviations: MPa, megapascal; SBS, shear bond strength; SD, standard deviation; TC, thermocycled.

Note: The superscripted letters indicate significant differences within the same column ( *p* < 0.05), the different lowercase letters indicate significant differences within the same line ( *p* < 0.05).

At the fractured zirconia/resin composite interface, the failure mode was detected using a stereomicroscope ( Table 3 ), which showed that the adhesive failure occurred in the no Tx group both of 24 hours and 10,000 cycles. Groups of CCB + SB2, GPU, CTB, and TNU exhibited predominantly mixed failures both before and after 10,000 cycles of thermocycling. OBU showed primarily mixed failures of 24 hours water storage, but adhesive failures increased after 10,000 cycles of thermocycling.

**Table 3:** Percentage of failure modes

Groups		Adhesive	Mixed	Cohesive
1. No Tx	24 hours	100	0	0
	10,000 TC	100	0	0
2. CCP + SB2	24 hours	30	70	0
	10,000 TC	30	70	0
3. GPU	24 hours 10,000 TC	10 40	90 60	0 0
4. CTB	24 hours 10,000 TC	30 40	70 60	0 0
5. OBU	24 hours 10,000 TC	30 70	70 30	0 0
6. TNU	24 hours 10,000 TC	40 40	60 60	0 0

Abbreviation: TC, thermocycling.

## Discussion

Zirconia is chemically inert. Surface modification with zirconia is necessary to achieve micromechanical retention and/or chemical bonding. 3 11 12 To achieve a durable zirconia/resin composite connection, the zirconia cleaning surface before the intraoral repair is important. The simplest method is air abrasion using alumina oxide particles, which is the most effective method for producing micromechanical retention of zirconia, as it improves the bond strength to zirconia. 13 14 It can also create a rough texture for zirconia surface, increase the zirconia surface for mechanical and chemical retention, and improve wettability. The air-abrasion should be conducted using 30 to 50 µm alumina oxide particles at 2.5 bars pressure in circular motion at a distance of 10 mm perpendicular to the zirconia surface for 10 to 20 seconds. 15 16

Phosphate and/or carboxylic monomers are included in commercially marketed zirconia surface modification agents. The commonly-used adhesives/primers include phosphate monomers, 10-MDP or GPDM, and carboxylic monomer, 4-META. These monomers are acidic bifunctional monomers with two functions, with the hydrophilic portion being the phosphate/carboxylic group and the hydrophobic portion being the vinyl group. Chemical bonds can be formed between the phosphate/carboxylic group and oxide layer of zirconia. The vinyl group can copolymerize with the resin monomer of the resin-based materials. Commercially manufactured universal adhesives and metal/zirconia primers contained acidic functional monomers that were efficient in improving the adhesion capacity of resin-based materials to zirconia. 17 18

In this research, we used one zirconia primer (CCP) and four universal adhesives (GPU, CTB, OBU, TNU) containing phosphate and/or carboxylic monomers. The GPU has 10-MDP and 4-MET containing monomers. CCP, CTB, and TNU have 10-MDP containing monomers. OBU has GPDM containing monomers. After each of the primers and universal adhesives had been applied to the zirconia surfaces, the SBS' significantly improved compared with the no Tx groups. GPU showed the highest initial bond strength. In contrast to other universal adhesives and CCP, GPU contains both phosphate monomer (10-MDP) and carboxylic monomer (4-MET). These two acidic functional monomers could enhance the initial bond strength of zirconia/resin composite. There was a need to differentiate between the effects of other universal adhesives and GPU on SBS. However, the initial bond strength for CCP + SB2, CTB, OBU, and TNU was not significant in between groups. Both phosphate monomers, 10-MDP and GPDM, were successful in increasing the initial bond strength of resin composite to zirconia. For this, the no Tx group served as the negative control, as it showed the lowest initial bond strength. Similarly, Han et al, Seabra et al, and Celik et al reported that the use of universal adhesives proved successful in increasing resin composite and zirconia adherence. 4 19 20

As part of the aging process, the SBS values of the GPU, OBU, and no Tx groups were lower after thermocycling for 10,000 cycles, but the SBS values of CCP + SB2, CTB, and TNU groups did not change significantly. Even after a long-term aging process, the bonding strength of zirconia/resin composites must be maintained. Thermocycling procedures were performed to compare the bond durability, according to aging. According to Gale et al, a cyclic method of 10,000 cycles per year may be sufficient to detect restorative failure. 10 This was because all universal adhesives and zirconia primer showed increased bonding strengths through an initial chemical bond with 4-MET and GPDM monomer bond, subsequently decreasing due to hydrolytic deterioration via 10,000 cycles of thermocycling procedures. For this reason, the 10-MDP monomer features a lengthy and hydrophobic spacer chain that improves bond strength and is stable even after 10,000 thermocycling cycles. 21 22 The 10-MDP has also been found to help improve and stabilize the bonding of resin composites to zirconia. The use of an extra-long chain hydrophobic spacer when using universal adhesives may improve the durability and resistance to deterioration of the zirconia/resin composite interface and also increase the long-term durability of resin composites. 23 24

After the SBS test, the mode of failure distribution confirmed the bond strength data. All of the samples for the no Tx group showed the lowest bond strength and exhibited adhesive failures both after 24 hours and after 10,000 cycles. The samples for the 10-MDP containing monomers showed predominantly mixed failures both before and after 10,000 cycles of thermocycling. The samples for the GPDM containing monomers presented primarily mixed failures after 24 hours, but adhesive failures increased after 10,000 cycles of thermocycling. Cohesive failures in the zirconia and resin composite were not found, which mentions that the weakest bond in the bound specimen occurred at the zirconia/resin composite interface.

## Conclusion

Within the scope of this study's limitations, the universal adhesives containing 10-MDP monomers performed best in terms of zirconia/resin composite interface' SBS both before and after thermocycling. Moreover, the SBS of all universal adhesives using 10-MDP monomers has not been substantially reduced by artificial aging. The universal adhesives containing 10-MDP monomers have the potential to improve the clinical method of repairing zirconia fractures, using resin composite, both in terms of initial bond and long-term stability.
